# Liquid Biopsy in Alzheimer’s Disease Patients Reveals Epigenetic Changes in the *PRLHR* Gene

**DOI:** 10.3390/cells12232679

**Published:** 2023-11-22

**Authors:** Mónica Macías, Blanca Acha, Jon Corroza, Amaya Urdánoz-Casado, Miren Roldan, Maitane Robles, Javier Sánchez-Ruiz de Gordoa, María Elena Erro, Ivonne Jericó, Idoia Blanco-Luquin, Maite Mendioroz

**Affiliations:** 1 Navarrabiomed, Hospital Universitario de Navarra, Universidad Pública de Navarra, IdiSNA, 31008 Pamplona, Spain; monica.macias.conde@navarra.es (M.M.); blancacha02@gmail.com (B.A.); amaya.urdanoz.casado@navarra.es (A.U.-C.); mroldana@navarra.es (M.R.); maitane.robles.solano@navarra.es (M.R.); javier.sanchez.ruizdegordoa@navarra.es (J.S.-R.d.G.); elena.erro.aguirre@navarra.es (M.E.E.); idoia.blanco.luquin@navarra.es (I.B.-L.); 2 Neurology Department, Hospital Universitario de Navarra, IdiSNA, 31008 Pamplona, Spain; jon.korroza@gmail.com (J.C.); ivonne.jerico.pascual@navarra.es (I.J.)

**Keywords:** Alzheimer’s disease, 450K array, DNA methylation, cell-free DNA, liquid biopsy, *PRLHR*, Prolactin releasing-peptide (PrRP)

## Abstract

In recent years, new DNA methylation variants have been reported in genes biologically relevant to Alzheimer’s disease (AD) in human brain tissue. However, this AD-specific epigenetic information remains brain-locked and unreachable during patients’ lifetimes. In a previous methylome performed in the hippocampus of 26 AD patients and 12 controls, we found higher methylation levels in AD patients in the promoter region of *PRLHR*, a gene involved in energy balance regulation. Our aim was to further characterize *PRLHR*’s role in AD and to evaluate if the liquid biopsy technique would provide life access to this brain information in a non-invasive way. First, we extended the methylation mapping of *PRLHR* and validated previous methylome results via bisulfite cloning sequencing. Next, we observed a positive correlation between *PRLHR* methylation levels and AD-related neuropathological changes and a decreased expression of *PRLHR* in AD hippocampus. Then, we managed to replicate the hippocampal methylation differences in plasma cfDNA from an additional cohort of 35 AD patients and 35 controls. The isolation of cfDNA from the plasma of AD patients may constitute a source of potential epigenetic biomarkers to aid AD clinical management.

## 1. Introduction

Alzheimer’s disease (AD) is a chronic neurodegenerative disorder with an enormous medical, economic, and social impact on our society. So far, multiple factors, such as environmental, biological, and genetic susceptibility, have been associated with the development of the disease. In recent years, scientific interest has been aroused by the influence that hormonal risk factors such as obesity and diabetes may have on AD, shaping a network of interaction that contributes to the inflammatory state occurring in this pathology [1]. Thus, anorexigenic and antidiabetic molecules, respectively, are gaining interest for their potential neuroprotective properties [2].

In age-related diseases such as AD, in which, in addition, environmental risk factors have been described, the study of epigenetic variants is of special interest. DNA methylation is the most widely studied epigenetic mechanism and involves the attachment of a methyl group to the 5-carbon position of a cytosine residue (5mC), usually occurring at cytosine-guanine dinucleotides (CpG). In this regard, candidate-gene approaches using human brain tissue samples have revealed differentially methylated variants in the promoter regions of several genes biologically relevant to AD [3]. Moreover, epigenome-wide studies have also revealed differentially methylated genes previously related to AD across different vulnerable brain regions in *postmortem* samples, such as *ANK1* [4,5], *HOXA* cluster [6], *ABCA7* [7], *TREM2* [8], or *MAMSTR* [9]. Interestingly, the human hippocampal methylome, a region particularly susceptible to AD, ref. [10], has been profiled, showing several DNA methylation marks on AD brains which are globally linked to neurogenesis [11]. These works have provided important data to enrich our understanding of AD pathogenesis. Even more, this AD-specific epigenetic information may become a source of candidate epigenetic biomarkers to aid AD clinical management [12,13]. However, these epigenetic markers remain locked in the brain tissue and, therefore, undetectable while the patient is alive. Hence, new approaches are required to gain access to that “brain-locked” valuable information, as is the case of liquid biopsy.

Liquid biopsy is a non-invasive method consisting of a blood test that allows for the isolation of cell-free DNA (cfDNA) circulating in the plasma [14]. Most applications of liquid biopsy so far are mainly based on detecting DNA sequence variability and have been proven useful in fields such as oncology [15] or prenatal genomic testing [16]. Beyond these applications, authors have profiled the tissue-specific methylation patterns of cfDNA and found increased brain-derived cfDNA in the serum of patients with multiple sclerosis or traumatic brain injury [17], which revealed that a significant proportion of circulating cfDNA may be derived from the affected brain tissue. Hence, this work opened up the door for new liquid biopsy applications in neurodegenerative diseases in which there is also a dysfunction of the blood–brain barrier, increasing its permeability and facilitating DNA from damaged neurons to reach the bloodstream [18,19]. Thereafter, several studies have pointed to differential methylation marks in plasma or serum cfDNA in neurodegenerative diseases such as the *RABEP1* and *RBFOX1* [20], *LHX2* [21], *MBP*, or *DUSP22* genes in AD patients [22], or *RHBDF2* in cfDNA from ALS patients [23]. Based on the above-mentioned evidence, we hypothesize that the cfDNA derived from AD damaged neurons may be isolated in the peripheral blood and further assessed to identify epigenetic biomarkers by using non-invasive liquid biopsy techniques.

In a previous methylome study performed on human AD hippocampus [11], we identified an increase in DNA methylation at the promoter region of *PRLHR* in AD patients. The *PRLHR* gene, also known as Prolactin-Releasing Hormone Receptor or GPR10, is located in chromosome 10q26.11. It encodes a seven-transmembrane domain receptor for prolactin-releasing hormone (PRLH, PrRP) a neuropeptide which, in addition to stimulating lactotrophs to secrete prolactin, is involved in energy balance regulation. Interestingly, PrRP has been proposed as a neuroprotective molecule due to its anorexigenic functions [24,25].

Here, following a translational approach, we aimed to test whether these hippocampal methylation changes in *PRLHR* could be observed in plasma cfDNA isolated from AD patients and controls so that we could gain access to epigenetic brain-locked information.

## 2. Materials and Methods

### 2.1. Hippocampal Samples

Brain hippocampal samples from 38 subjects (26 AD patients and 12 controls) were provided by Navarrabiomed Brain Bank. After death, half-brain specimens from donors were cryopreserved at −80 °C. A neuropathological examination was completed following the usual recommendations [26]. Assessment of β-amyloid deposition was carried out via the immunohistochemical staining of paraffin-embedded sections (3–5 μm thick) with a mouse monoclonal (S6F/3D) anti β-amyloid antibody (Leica Biosystems Newcastle Ltd., Newcastle upon Tyne, UK), (dilution 1:200). Evaluation of neurofibrillary pathology was performed with a mouse monoclonal antibody anti-human PHF-TAU, clone AT-8 (Tau AT8) (Innogenetics, Gent, Belgium), (dilution 1:1000), which identifies hyperphosphorylated tau (p-tau) [27]. The reaction product was visualized using an automated slide immunostainer (Leica Bond Max) with Bond Polymer Refine Detection (Leica Biosystems, Newcastle Ltd.). AD staging was performed by using the ABC score according to the updated National Institute on Aging-Alzheimer’s Association guidelines [28].

To avoid any spurious findings related to multi-protein depositions, only AD cases with pure p-tau and β-amyloid deposits were included in the study, and controls were free of any pathological protein aggregates. This approach maximizes the chances of finding true molecular associations with AD, although it reduces the number of older controls.

For the quantification of both AD neuropathological hallmarks in the AD hippocampal samples, we used a method detailed in a previous article [29]. After performing immunostaining with anti β-amyloid and anti p-tau antibodies, the hippocampal sections were examined and representative images were analyzed with ImageJ software v1.52d to obtain an average quantitative measure for each section and patient (Supplementary Figure S1).

### 2.2. Validation of PRLHR Methylation Changes in Hippocampal Samples Using Bisulfite Cloning Sequencing

In this study, we used our previous DNA methylation microarray data obtained by the Infinium Human Methylation 450K BeadChip (450K array) [11]. For the validation of *PRLHR* differential methylation levels, we employed bisulfite cloning sequencing, traditionally considered to be the gold standard technique for locus-specific DNA methylation studies [30,31]. Genomic DNA was isolated from the hippocampal samples using the phenol-chloroform method [32]. An amount of 500 ng of DNA from each sample was bisulfite converted using the EpiTect Bisulfite Kit (Qiagen, Redwood City, CA, USA). A region of 321 bp spanning 19 CpG dinucleotides, including the CpG assayed in the 450K array (cg19403534), was explored. The MethPrimer tool v1 was used for primer pair sequence design [33], as shown in Supplementary Table S1. PCR products were cloned using the TopoTA Cloning System (Invitrogen, Carlsbad, CA, USA), and a minimum of 10–12 independent clones were sequenced for each sample. QUMA software v1.1.13 was used to generate methylation graphs [34].

### 2.3. PRLHR mRNA Expression Analysis Using Real-Time Quantitative PCR (RT-qPCR)

The total RNA was extracted from the hippocampal samples using the RNeasy Lipid Tissue Mini kit (QIAGEN, Redwood City, CA, USA), according to the manufacturer’s instructions. Genomic DNA was removed with recombinant DNase (TURBO DNA-free™ Kit, Ambion, Inc., Austin, TX, USA). A NanoDrop spectrophotometer was used to evaluate both the RNA concentration and purity. Only the RNA samples showing a minimum quality index (260 nm/280 nm absorbance ratios between 1.8 and 2.2 and 260 nm/230 nm absorbance ratios higher than 1.8) were included in the study. Complementary DNA (cDNA) was reverse-transcribed from 1500 ng of total RNA with SuperScript^®^ III First-Strand Synthesis Reverse Transcriptase (Invitrogen, Carlsbad, CA, USA) after priming with oligo-d (T) and random primers. RT-qPCR reactions were performed in triplicate with Power SYBR Green PCR Master Mix (Invitrogen, Carlsbad, CA, USA) in a QuantStudio 12K Flex Real-Time PCR System (Applied Biosystems, Foster City, CA, USA) and repeated twice within independent cDNA sets. The Real Time PCR tool (IDT, Coralville, IA, USA) was used to design primer pair sequences (Supplementary Table S1). The relative expression level of *PRLHR* mRNA in each sample was calculated as previously described [35] and the geometric means of the *ACTB* and *GAPDH* genes were used as reference for the expression values’ normalization.

### 2.4. Plasma Samples

Next, we conducted a case–control study including 70 subjects (35 AD patients and 35 cognitively healthy controls) to explore if the *PRLHR* methylation differences detected in the hippocampus could be also identified in plasma cfDNA from AD patients and controls.

Patients were prospectively recruited from the Dementia Unit of University Hospital of Navarra (tertiary hospital) from March 2019 to December 2021. Alzheimer’s disease was diagnosed according to the guidelines of the National Institute on Aging and Alzheimer’s Association (NIA-AA 2018) [36] and classified according Global Deterioration Scale (GDS ≥ 4) [37]. Controls were recruited from healthy relatives and volunteers matched for age and sex with the following features: no clinical manifestation of dementia or other neurodegenerative disease and no tumoral disease in at least last five years. The Ethics Committee approved the study and all the participants signed an informed consent form.

Peripheral blood samples were collected from each subject via venipuncture in 10 mL PAXgene^®^ Blood DNA Tubes and centrifuged at 1900× *g* at room temperature for 15 min within an hour. Plasma was transferred to 2 mL plastic tubes, centrifuged for a second time at 20,000× *g*, and stored at −80 °C until further analysis.

### 2.5. PRLHR Methylation Measurement in Plasma cfDNA Using Pyrosequencing

cfDNA was isolated from 2 mL of plasma by using QIAmp Circulating Nucleic Acid Kit (QIAGEN, Redwood City, CA, USA) according to the manufacturer’s instructions. An amount of 200 ng of cfDNA from each sample was bisulfite converted using the EpiTect Bisulfite Kit (Qiagen, Redwood City, CA, USA). Primers to amplify and sequence a target region in the *PRLHR* gene were designed with PyroMark Assay Design version 2.0.1.15 (Qiagen Redwood City, CA, USA), and PCR reactions were carried out on a Veriti^TM^ Thermal Cycler (Applied Biosystems, Foster City, CA, USA) (Supplementary Table S1). Next, 20 μL of biotinylated PCR product was immobilized using streptavidin-coated sepharose beads (GE Healthcare Life Sciences, Piscataway, NJ, USA) and 0.3 μM of sequencing primer was annealed to purified cfDNA strands. Pyrosequencing was performed using the PyroMark Gold Q96 reagents (Qiagen) on a PyroMark™ Q96 ID System (Qiagen). For each CpG studied (CpG1 and CpG2), methylation levels were expressed as percentage of methylated cytosines over the sum of total cytosines. The EpiTect PCR Control DNA Set (Qiagen) was used as unmethylated and methylated DNA controls for the pyrosequencing reaction.

### 2.6. Statistical Analysis

A statistical analysis was performed with SPSS v25 (IBM Corp., Armonk, NY, USA). Prior to differential analysis, the continuous variables were tested for normal distribution using one-sample Kolgomorov–Smirnov test and normal quantil-quantil (QQ) plots. The data are represented as mean and standard deviation (SD) if normal distribution is followed, or as median and interquartile range (IQR) otherwise. The Mann–Whitney U test was used to evaluate the statistical differences between two bisulfite cloning sequencing groups. *PRLHR* methylation differences performed using pyrosequencing were assessed by Student’s *t* test. *PRLHR* methylation levels were correlated with AD-related pathology parameters using Spearman correlation for non-parametric data. A logistic regression model (ENTER method) was fit to assess the independent association of the *PRLHR* methylation levels in the hippocampal samples and the plasma cfDNA with AD status, using sex and age as covariates, and the odds ratio (OR) was calculated. The diagnostic performances of the DNA from the hippocampal samples and cfDNA *PRLHR* methylation levels were evaluated with receiver operating characteristic (ROC) curves. The area under the curve (AUC) was calculated and optimum cut-off points of each matrix was selected based on its sensitivity and specificity. The data distribution for each analysis was first assessed. Results that were outside the following ranges: Q1 − 3 × IQR or Q3 + 3 × IQR were considered as extreme outliers and were therefore excluded for a downstream analysis. The significance level was set at *p*-value < 0.05. GraphPad Prism version v9 for Windows (GraphPad Software, La Jolla, CA, USA) was used to draw graphs.

## 3. Results

### 3.1. Samples Characteristics

In order to validate the methylation levels of the *PRLHR* gene from 450K array results [11], we used eight hippocampal samples. The demographic and neuropathological characteristics of these eight samples, including age, sex, ABC score, ABC scale, Braak stage, and *postmortem* interval (PMI), are shown in Supplementary Table S2.

For an additional liquid biopsy study, 35 AD patients and 35 sex- and age-matched healthy volunteers were recruited at the Neurology Department-University Hospital of Navarre. The demographic and clinical characteristics of the subjects, including age, sex, GDS, and Mini Mental State Examination (MMSE), are summarized in Table 1. There were no significant differences in age or sex between the AD patients and controls. As expected, the cognitive function scores were significantly different in the AD patients compared to the controls (*p*-value < 0.001) (Table 1).

### 3.2. DNA Methylation Levels in PRLHR Are Increased in Hippocampus of AD Patients Compared to Controls

**Figure 1:**
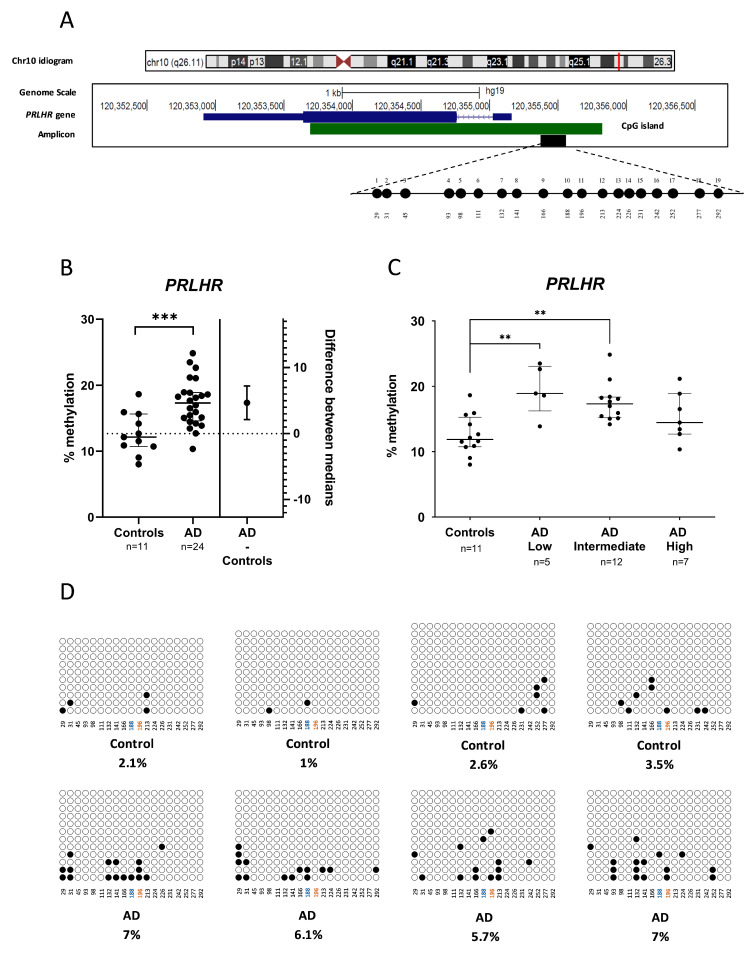
DNA methylation levels in the promoter region of *PRLHR* gene in hippocampus from Alzheimer’s disease (AD) and controls. (**A**) The figure shows the genomic position of the amplicon (black box) validated using bisulfite cloning sequencing which contains the CpG assayed by the Infinium Human Methylation 450K BeadChip array within the promoter region of *PRLHR* gene. An example of the 19 CpGs composing the amplicon fully methylated (black circles) is shown. Numbers below indicate each CpG position within the amplicon in base pairs. *PRLHR* is located on the long arm of chromosome 10 (chr10: 120, 352, 916–120, 355, and 160). The CpG island is represented by a green box as shown in the UCSC Genome Browser. (**B**) Dot-plot chart representing 450K methylation levels for *PRLHR* hippocampal samples. As seen in the figure, a significant increase in DNA methylation was identified between AD patients and controls. (**C**) Dot-plot chart representing 450K methylation levels for *PRLHR* according to ABC scale. Horizontal lines represent median methylation values and interquartile range for each group. (**D**) Representative examples of bisulfite cloning sequencing validation for the amplicon containing the CpGs are shown. Black and white circles represent methylated and unmethylated cytosines, respectively. Each column indicates every CpG site in the examined amplicon, and each row represents an individual DNA clone. CpG1 (blue) and CpG2 (orange) assessed by pyrosequencing are represented. *** *p*-value < 0.001, ** *p*-value < 0.01 (Mann–Whitney U test).

First, we aimed to validate the DNA methylation results obtained in the 450K array analysis [11] in the promoter region of the *PRLHR* gene. This region is located at the 5′ end of the gene and contains a CpG island of 2,129 bp (chr10:120353692-120355821), as shown by the UCSC Genome Browser website [38] (Figure 1A).

The results from our previous 450K array performed in human hippocampalsamples showed a 1.5-fold increase in the methylation in AD patients with respect to controls [AD median = 17.33% (IQR = 14.62–18.92) and controls median = 11.61% (IQR = 10.71–14.19; *p*-value < 0.001)] (Figure 1B). Moreover, we observed a significant increase in *PRLHR* methylation levels when classifying the AD patients according to their ABC scale. Namely, *PRLHR* methylation was increased in both low [median = 18.93% (IQR = 16.24–23.06)] and intermediate [median = 17.32% (IQR = 15.20–18.37)] AD patients with respect to controls (*p*-value < 0.01, for both comparisons) (Figure 1C). We extended the methylation mapping in an amplicon overlapping the *PRLHR* promoter region by using bisulfite cloning sequencing. In line with the previous 450K array results, we found that the average DNA methylation levels for the whole amplicon were significantly increased in the AD patients compared to the controls (6.45 ± 0.65 % vs. 2.30 ± 1.04 %; *p*-value = 0.001) (Figure 1D).

Although the previous 450K array analysis yielded *PRLHR* statistical differential methylation after adjusting for age, a correlation analysis pointed to an existing association between these two variables (r = 0.589; *p*-value < 0.001; Spearman correlation). We therefore built a univariate general linear model adjusted by age as a potential confounder, resulting in only the presence of AD explaining *PRLHR* methylation values (*p*-value < 0.05) while age did not (*p*-value = 0.157)

Next, a binary logistic regression model was performed to test whether the CpG methylation levels found in the 450K array were independently associated with AD status (control = 0; AD = 1). Age and sex were included into the model in order to adjust for potentially confounding variables, since there were significant age differences between the control and AD groups (*p*-value < 0.05). As shown in Table 2, *PRLHR* methylation levels in the hippocampal samples remained an independent predictor of AD status after adjusting for age and sex (*p*-value < 0.05) with an odds ratio (OR)= 1.65 (CI 95% = 1.03–2.63) (*p*-value < 0.05).

### 3.3. PRLHR Gene Expression Is Decreased in AD Hippocampus Compared to Controls

**Figure 2:**
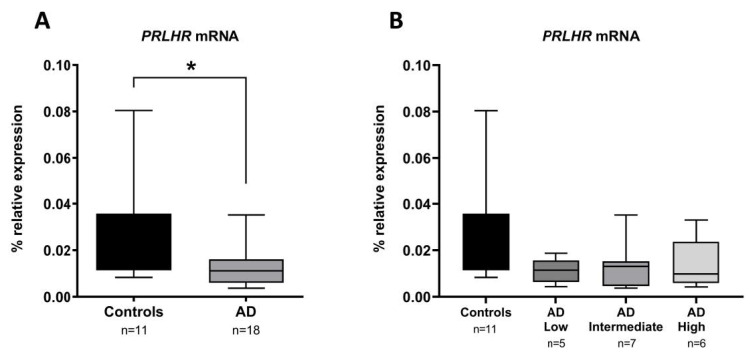
*PRLHR* expression profiling in hippocampus of Alzheimer’s disease (AD). (**A**) The box plot graphs show *PRLHR* mRNA levels in the hippocampus from AD patients compared to controls and (**B**) when stratifying AD patients according to ABC scale. * *p*-value < 0.05 (Mann-Whiney U test).

Then, we aimed to evaluate whether the *PRLHR* gene was differentially expressed in the AD hippocampus when compared to the controls. For that purpose, we measured the *PRLHR* mRNA levels using RT-qPCR. As shown in Figure 2A, the *PRLHR* mRNA levels in the hippocampus were significantly decreased 2.25-fold in AD cases compared to controls (*p*-value < 0.05). Nevertheless, when stratifying the patients across the ABC scale, we no longer found significant differences according to AD severity (*p*-value = 0.131) (Figure 2B).

### 3.4. Correlation of PRLHR Methylation Levels in Hippocampal Samples with β-Amyloid and p-tau Deposits

**Figure 3:**
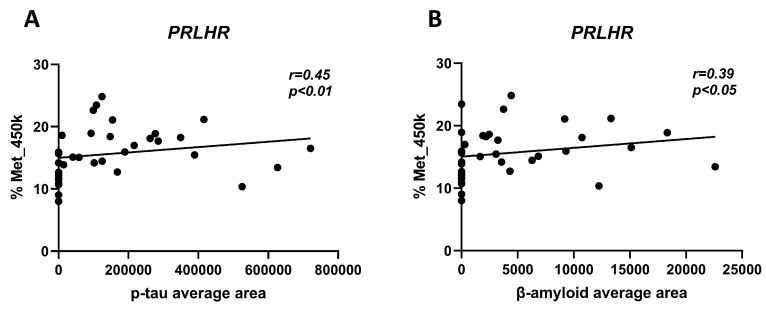
Scatter plots graphs of Spearman correlation analysis between DNA methylation levels of 450K and p-tau burden (**A**) and β-amyloid deposition (**B**). A significant positive correlation was found between *PRLHR* methylation and p-tau (r = 0.45; *p*-value < 0.01) and β-amyloid (r = 0.39; *p*-value < 0.05) (*n* = 24).

We wanted to ascertain a possible correlation between *PRLHR* methylation levels and AD-related neuropathological changes in the hippocampal samples. The average area of both β-amyloid deposits and p-tau burden were quantitatively measured and recorded from the hippocampus sections, as described in the Section 2, with ImageJ software v1.52d [39]. Interestingly, we observed a significant Spearman correlation between both the 450K array results and the average area of the p-tau burden (r = 0.457; *p* = 0.006) and average area of β-amyloid deposits (r = 0.385; *p*-value < 0.05) (Figure 3). These results suggest that the increase in methylation parallels the molecular changes induced by β-amyloid and p-tau deposition.

### 3.5. Methylation Differences in PRLHR Can Be Detected in Plasma cfDNA of AD Patients Compared to Controls

In this study, we explored the feasibility of applying liquid biopsy techniques to assess AD-related methylation changes in cfDNA from living patients. Thus, we opted to use a less labor-intensive method more suitable in a clinical setting, such as the pyrosequencing technique.

**Figure 4:**
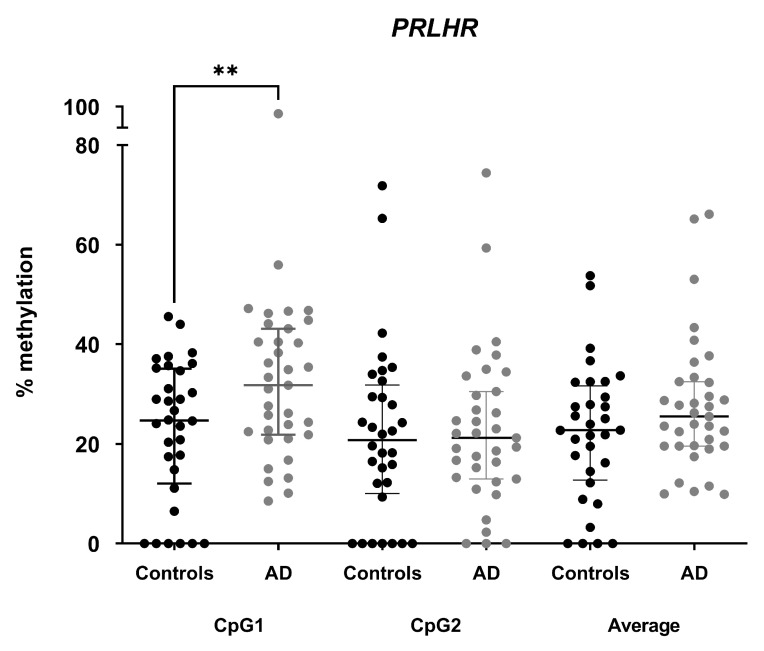
*PRLHR* cfDNA methylation levels in plasma samples. Dot-plot charts showing methylation levels for CpG1 and CpG2 and in average between Alzheimer’s disease (AD) patients (*n* = 35) and controls (*n* = 32). Horizontal lines represent median methylation values and interquartile range for each group. ** *p*-value < 0.01 (Student’s *t* test).

The amounts of cfDNA ranged from 0.68 ng/µL to 26.6 ng/µL and no significant differences were found between groups (*p* = 0.445). We furthermore measured cfDNA methylation levels of CpG1 and CpG2 of the *PRLHR* promoter region. Three controls failed to show an amplification prior to pyrosequencing, so they were discarded from the analysis. The pyrosequencing analysis revealed statistically significant higher *PRLHR* cfDNA methylation levels in AD with respect to controls in CpG1 (32.7 ± 16.2% vs. 22.7 ± 14.1%; *p*-value < 0.01) and no significant differences in CpG2 (22.7 ± 15.8% vs. 21.7 ± 17.6%; *p*-value = 0.805) (Figure 4). Neighboring CpG sites are more likely to have a similar methylation pattern [40]. As expected, we observed a positive correlation between the methylation levels of CpG1 and CpG2 (r = 0.427; *p*-value < 0.001; Spearman correlation).

Next, we performed a binary logistic regression model to test whether the CpG methylation in cfDNA was an independent predictor of AD (control = 0; AD = 1). Age and sex were included in the model in order to adjust for potentially confounding variables. As shown in Table 2, the *PRLHR* methylation levels in plasma cfDNA remained an independent predictor of AD status after adjusting for age and sex (*p*-value < 0.05), with an odds ratio (OR)= 1.048 (CI 95% = 1.008–1.089; *p*-value < 0.05).

To explore the performance of the *PRLHR* methylation levels in plasma cfDNA for AD diagnosis, an a ROC analysis was performed (Supplementary Figure S2). The AUC was 0.662 (CI 95% = 0.532–0.791; *p*-value < 0.05) and the optimal cutoff point to differentiate between AD patients and controls was 20.96% (sensitivity = 0.80; specificity = 0.41).

### 3.6. Correlation of PRLHR Methylation Levels in cfDNA with Clinical Parameters

We also wanted to test the relationship between the cfDNA methylation levels of *PRLHR* in our set of samples with clinical parameters. A significant correlation was found between the cfDNA methylation percentage measured with pyrosequencing and age in CpG2 (r = −0.242; *p*-value < 0.05), and no correlation was shown in CpG1 (r = 0.038; *p*-value = 0.757; Spearman correlation for both tests).

We furthermore compared the *PRLHR* methylation in cfDNA with cognitive status and dementia scale. We found a correlation between the *PRLHR* methylation levels in the cfDNA for CpG1 and GDS (r = 0.270; *p*-value < 0.05) and a correlation trend, although it did not reach significance with MMSE (r = −0.258; *p*-value = 0.051; Spearman correlation for both tests).

## 4. Discussion

In this study, we validated the previous methylome results in the promoter region of the *PRLHR* gene in the AD hippocampus. Furthermore, we found that the *PRLHR* methylation levels showed a positive correlation with both the β-amyloid deposits and p-tau burden quantified in hippocampal samples. In addition, a significant reduction in *PRLHR* gene expression was found in the AD hippocampus. Moreover, we were able to replicate these methylation differences in cfDNA from the peripheral blood of an additional cohort of AD patients and controls, demonstrating the feasibility of employing liquid biopsy techniques to detect methylation differences in the plasma cfDNA of AD patients and controls.

Our results indicate that the promoter region of the *PRLHR* gene is differentially methylated in the AD hippocampus compared to controls. Moreover, methylation differences appear to be present since early stages, according to the ABC scale. We extended the methylation mapping of this region and observed the same differences in DNA methylation. Interestingly, we also found decreased expression of *PRLHR* in the AD hippocampus with respect to the controls. Since the methylation of CpG islands located in promoter regions traditionally leads to the silencing of gene expression, we hypothesize that the increased *PRLHR* methylation levels found in AD patients may induce the downregulation of the *PRLHR* receptor expression in the hippocampus of AD patients. Moreover, we found a strong significant correlation between the methylation of *PRLHR* levels in the hippocampus and age at death for our study cohort (r = 0.589; *p*-value < 0.001). These two aforementioned findings could be explained by the decreased expression of neuroprotective genes, as well as known epigenome changes appearing during aging [41]. In this regard, the *PRLHR* gene has been previously proposed as a candidate gene for the epigenetic drift occurring during aging [42,43,44]. Nevertheless, differences in *PRLHR* methylation remained significant in our study cohort after adjusting for age. These results support the idea that epigenetic changes during aging might also contribute to neurodegeneration and may represent potential biomarkers for AD.

Another important point to highlight is the relevance of the adult hippocampal neurogenesis taking place in the aging process, pointing to impaired neurogenesis as a potentially crucial mechanism underlying neurodegeneration in AD [45]. Moreover, an enrichment and functional analysis of differentially methylated genes in the AD hippocampus revealed a strong link to neurogenesis [11]. Interestingly, we found that the PRLHR natural ligand has been proven to be related to this process. Zmeškalová et al. observed an increased viability of human neuroblastoma cell line SH-SY5Y cells when treated with PrRP, which reinforced the idea of its neuroprotective capacity [46]. The study of Mráziková et al. pointed to an increase in synaptogenesis and neurogenesis in the brains of animals treated with the administration of palmitoylated PrRP analogs, leading to a reduction in neurodegeneration with an increase in insulin and leptin signaling [47]. We hypothesize that the PrRP receptor may also be necessarily involved in this neurogenesis process.

The *PRLHR* gene encodes for the GPR10, a receptor widely expressed in the brain by which PrRP exerts its actions. PrRP was initially identified as a peptide that promoted prolactin (PRL) release from rat anterior pituitary cells [24]. However, in subsequent years, it has been demonstrated to play an important role as a neuromodulator in the central nervous system due to its strong anorexigenic properties [48,49]. Obesity and type 2 diabetes have been extensively characterized as risk factors for late-onset AD. The association existing between them and AD is such that it has led to the latter being commonly referred to as “diabetes of the brain” or “type 3 diabetes" [50,51]. Therefore, special attention has been devoted over past years to food- intake-lowering peptides employed for the treatment of both diabetes and obesity as PrRP as potential disease-modifying drugs for AD [52].

Several animal models of AD disease have already proven that lipidized PrRP analogs exhibit neuroprotective properties, as well as a better ability to cross the blood–brain barrier. Holubova et al. observed the neuroprotective properties of a lipidized PrRP analog in a mice model of amyloidosis throughout a significant reduction in β-amyloid plaque burden, in microglia-mediated inflammation, in tau phosphorylation, and an increase in adult neurogenesis [53]. Spolcova et al. showed a strong connection between obesity-related hippocampal impaired-insulin signaling and tau hyperphosphorylation occurring in the brain [25]. For these reasons, PrRP lipidized analogs have been postulated as compounds of great interest in the attenuation of the main features occurring in AD [54]. Despite the above-mentioned evidence on PrRP analogs in AD animal models, little is known about GPR10 activation and how it is implicated in energy balance and neuroprotection, which made us wonder whether the PrRP receptor may also be implicated in AD onset. Most interestingly, we observed a positive correlation between *PRLHR* methylation in our set of hippocampal samples and both principal AD neuropathological hallmarks, β-amyloid deposits (r = 0.385; *p*-value < 0.05), and p-tau burden (r = 0.457; *p*-value = 0.006). In our hands, these data support the idea that DNA methylation at the promoter region of the *PRLHR* receptor and the likely decline of its expression could be involved, in conjunction with PrRP, in the buildup of AD signature proteins in the hippocampus, a brain region most vulnerable to AD. In any case, however, the exact underlying molecular mechanism should be elucidated.

Liquid biopsy has been postulated in recent years as a potential surrogate for tissue molecular profiling in several types of cancer. In the case of neurodegenerative diseases such as AD, in which brain tissue is not accessible while patients are alive, liquid biopsy opens the way to more personalized medicine, as well as to a living diagnosis even in the early preclinical and prodromal stages. Peripheral blood mononuclear cells (PBMCs) have been proposed as another potential non-invasive source for DNA methylation biomarkers. Indeed, a number of works have been conducted showing parallels between DNA methylation marks in the brain and PBMCs [55,56]. For instance, Monti et al. explored *PSEN1* methylation differences simultaneously in brain and peripheral PBMCs, proposing the search for *PSEN1* methylation as a potential biomarker for the disease [57]. Nevertheless, the search for epigenetic biomarkers for neurodegenerative diseases is still only emerging.

In our study cohort, we managed to obtain cfDNA from the plasma of all patients and controls, although this amount of cfDNA varied considerably among individuals, thus probably interfering in the experiment output, as already addressed by other groups [58]. In this regard, we were not able to amplify this region in the cfDNA from three controls. While higher concentrations of plasma CNAs (circulating nucleic acids) have been reported for AD patients, pointing to a strong association with AD or cognitive impairment [21], in our hands, the cfDNA concentrations from AD patients were not significantly higher compared to controls. This is in line with previously published studies reporting overlapping concentrations in pathological conditions with those observed for healthy individuals [59]. Moreover, we verified that cfDNA concentrations did not correlate with methylation levels, demonstrating that the degree of methylation detected was not depending on the cfDNA amount.

AD diagnosis is mainly based on clinical symptoms such as progressive cognitive decline measured by cognitive test scores, along with neuroimaging and CSF biomarkers. Very interestingly, we further found a significant correlation between the *PRLHR* methylation levels in cfDNA and GDS (r = 0.270; *p*-value < 0.05) and a correlation trend with MMSE (r = −0.258; *p*-value = 0.051). These results may be explained by the energy balance dysregulation taking place in AD pathophysiology.

Currently, it remains difficult to detect methylation differences sensitive enough in cfDNA originating from brain cells among background cfDNA [13]. In health, plasma cfDNA mainly results from the apoptosis of haematopoietic cells. However, during disease processes, a significant proportion of cfDNA may originate from the affected tissue [60]. In this regard, although the brain origin of cfDNA in our study cannot be properly demonstrated, we hypothesize that a higher proportion of cfDNA derived from dying CNS cells might cross the altered blood–brain barrier and be isolated from the peripheral blood in AD patients [18,19,23]. It is worth noting that we managed to replicate the brain tissue results in the plasma cfDNA isolated from an additional cohort of AD patients. Moreover, the *PRLHR* methylation levels in liquid biopsy remained to show potential diagnostic applications founded on both a significant logistic regression model and ROC analysis. These findings support that *PRLHR* methylation levels may be a peripheral epigenetic biomarker mimicking brain tissue molecular changes worth exploring with liquid biopsy procedures. In any case, the ROC curve for the *PRLHR* methylation levels was of a poor discrimination capability. This is not discouraging, since the predictive value of methylation marks is likely to be useful through the construction of a panel of multiple biomarkers rather than the use of a single epigenetic biomarker.

A limitation of this study is that it focuses only on methylation at CpG sites and does not take into account the methylation that occurs at cytosines that do not precede guanine nucleotides. This epigenetic mechanism known as non-CpG or CpH methylation has been linked to complex brain functions in mammals [61]. Further studies should be conducted to explore changes in CpH methylation as potential biomarkers for neurodegenerative diseases.

## 5. Conclusions

We provide added insight on DNA methylation in *PRLHR*, a gene involved in energy balance regulation, a process closely related to AD development. Changes in DNA methylation suggest a role for the PrRP receptor in the AD brain, and what is more, we managed to replicate the *PRLHR* methylation hippocampal results in the plasma cfDNA of living AD patients. The liquid biopsy technique would provide access to AD-specific epigenetic information in a non-invasive way during patients’ lifetimes. Further studies in larger and independent cohorts may lead to more knowledge about this finding.

## Figures and Tables

**Table 1:** Plasma sample set analyzed using pyrosequencing. The table shows the phenotypical features of the subjects included in the study.

Phenotypical Features	Controls (*n* = 35)	AD Patients (*n* = 35)	*p*-Value
Median (IQR)			
Age (years)	77 (72–79)	78 (773–783)	0.154
MMSE	30 (29–30)	22 (19–26)	<0.001
GDS	1 (1–1)	4 (4–4)	<0.001
cfDNA conc (ng/µL)	2.1 (1.0–5.1)	1.8 (0.7–5.5)	0.445
N (%)			
Sex			0.811
Female	17 (49)	18 (51)	
Male	18 (51)	17 (49)	

**Table 2:** Adjusted logistic regression model to predict AD status in hippocampus and cfDNA. AD status (control = 0; AD = 1) was considered as the dependent variable. *PRLHR* methylation levels in hippocampus and plasma were included as covariates. Age and sex were included as covariates in the logistic regression model. B: regression coefficient, OR: odds ratio, CI: confidence interval, * *p*-value < 0.05.

	Variable	B	Wald	*p*-Value	OR	95% C.I. for OR
Hippocampus	MET_*PRLHR*_450K	0.50	4.37	0.04 *	1.65	1.03–2.63
	Age	−2.28	3.79	0.05	0.10	0.01–1.02
	Sex (female)	−0.65	0.28	0.60	0.52	0.05–5.92
	Constant	−4.89	2.05	0.15	0.01	
cfDNA	MET_*PRLHR*_CpG1	0.05	5.69	0.02 *	1.05	1.01–1.09
	Age	0.06	1.31	0.25	1.06	0.96–1.16
	Sex (female)	0.39	0.53	0.47	1.47	0.52–4.17
	Constant	−5.64	2.20	0.14	0.01	

## Data Availability

The data presented in this study are available on request from the corresponding author.

## Supplementary Materials

The following supporting information can be downloaded at: https://www.mdpi.com/article/10.3390/cells12232679/s1. Supplementary Table S1. Primer pairs employed for pyrosequencing and bisulfite cloning sequencing. Supplementary Table S2. Brain sample set analyzed by bisulfite cloning sequencing. Supplementary Figure S1. Quantitative assessment of β-amyloid (A,B) and p-tau deposits (C,D) in hippocampus. Supplementary Figure S2. ROC curve analysis of *PRLHR* methylation levels in plasma cfDNA in discriminating Alzheimer’s disease (AD) patients from controls.
