# Supplementary material for: Liquid Biopsy in Alzheimer’s Disease Patients Reveals Epigenetic Changes in the PRLHR Gene
Source: Cells. 2023 Nov 22;12(23):2679. doi: 10.3390/cells12232679 (PMC10705731; doi:10.3390/cells12232679)

Supplementary Figure S1. Quantitative assessment of β-amyloid (A-B) and p-tau deposits (C-D) in hippocampus.

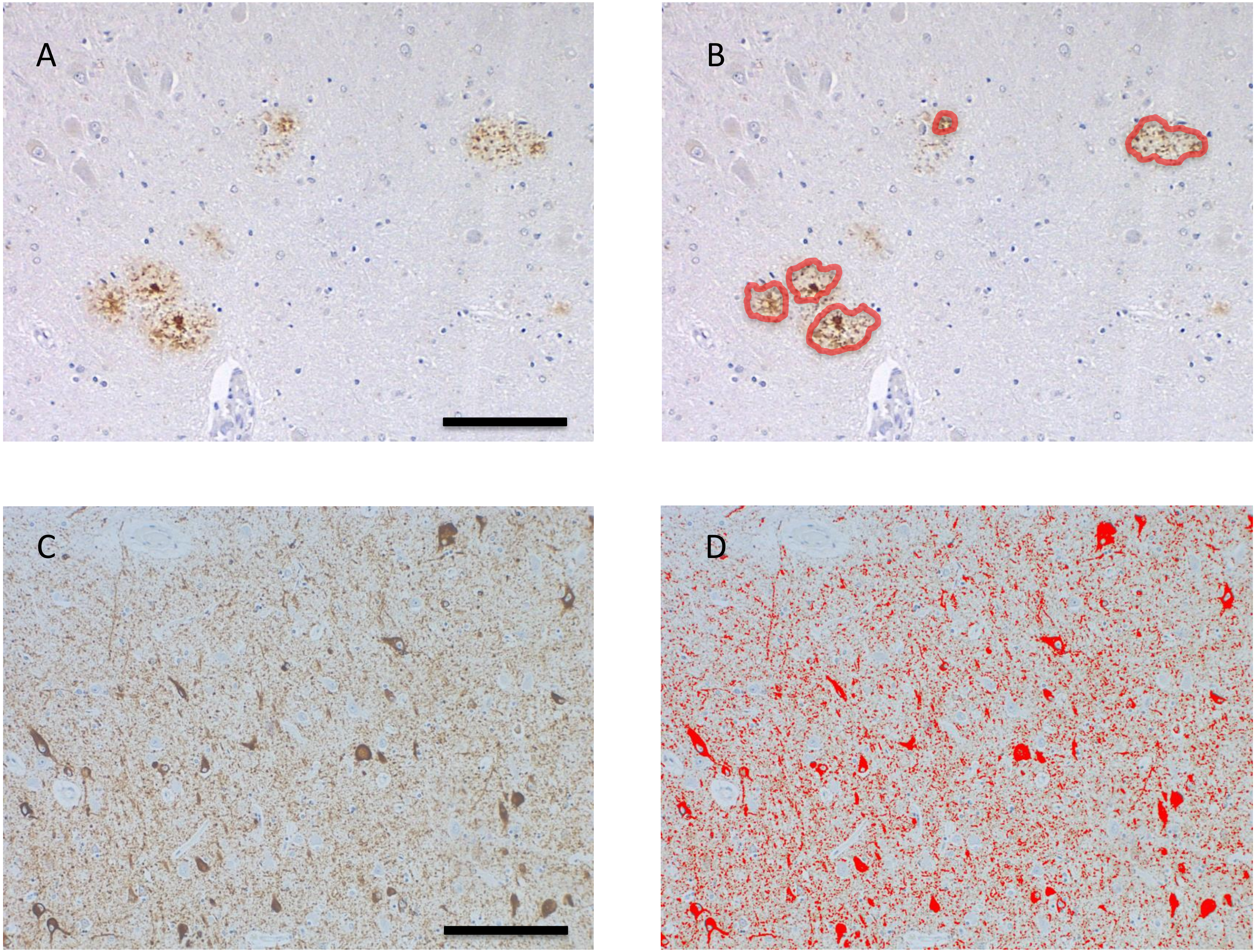

Supplement: Supplementary file 1 [file cells-12-02679-s001.zip › PRLHR_Supplementary Figure1_revised.pdf]
