# Supplementary material for: Liquid Biopsy in Alzheimer’s Disease Patients Reveals Epigenetic Changes in the PRLHR Gene
Source: Cells. 2023 Nov 22;12(23):2679. doi: 10.3390/cells12232679 (PMC10705731; doi:10.3390/cells12232679)
Supplement: Supplementary file 1 [file cells-12-02679-s001.zip › PRLHR_Supplementary Table1_revised.pdf]

Supplementary Table S1. Primer pairs employed for pyrosequencing and bisulfite cloning sequencing. Bp: base pair; Tm: melting temperature

Primer	Application	Amplicon size (bp)	Tm1	Forward Primer	Tm2	Reverse Primer
PRLHR _pyro	PCR pyrosequencing	170	61.2	AGGGGTAGGTAAATTTGGTTAGAGTAG	61.6	[Btn]CCTTCCCTCCTAAAACTACACC
PRLHR _seq			43.5	GGTTTTTGGAGGGTTA		
PRLHR _bis	Bisulfite cloning sequencing	321	59.5	TTTTTATGTATGGAAAATAGGGGTG	59.6	AACCCCCAACTACATTACAATCTC
PRLHR _q	qPCR	82	61.1	CTCTCCAAACCCCACTCC	61.2	GCAGCCCAGAAAATAAGTCAG
