# Supplementary material for: Liquid Biopsy in Alzheimer’s Disease Patients Reveals Epigenetic Changes in the PRLHR Gene
Source: Cells. 2023 Nov 22;12(23):2679. doi: 10.3390/cells12232679 (PMC10705731; doi:10.3390/cells12232679)
Supplement: Supplementary file 1 [file cells-12-02679-s001.zip › PRLHR_Supplementary Table2_revised.pdf]

Supplementary Table S2. Brain sample set analyzed by bisulfite cloning sequencing. The table shows the characteristics of the samples included in the validation step. No.: Number; AD: Alzheimer's disease; PMI: post mortem interval; h: hours; NA: not aplicable

No.	Diagnosis	Braak stage	ABC score	ABC scale	Age at death (years)	Sex	PMI (h)
1	Control	0	Control	NA	46	Female	7
2	Control	0	Control	NA	41	Male	3.5
3	Control	0	Control	NA	26	Male	6.2
4	Control	0	Control	NA	61	Male	8
5	AD	II	A1B1C1	Low	66	Female	1.4
6	AD	III	A1B2C1	Low	96	Female	1.5
7	AD	IV	A2B2C2	Int	97	Female	NA
8	AD	V	A3B3C2	High	91	Male	5
